# Development of a predictive model for depressive symptoms in type 2 diabetes mellitus patients under community management: Based on visual function index

**DOI:** 10.1002/ibra.70014

**Published:** 2026-02-12

**Authors:** Rong‐song Sun, Sheng‐xu Huo, Tian‐lin Zhang, Hao‐jiang Ying, Jun‐hua Wang, Li‐na Xu, Xin‐lin Luo, Yi Yang, Yuan‐dong Hu

**Affiliations:** ^1^ School of Public Health, the Key Laboratory of Environmental Pollution Monitoring and Disease Control, Ministry of Education Guizhou Medical University, Guian New Area China; ^2^ College of Computer Science and Electronic Engineering Hunan University Changsha China; ^3^ Health Management Department Guizhou Nursing Vocational College Guiyang China; ^4^ Department of Psychology Soochow University Suzhou China; ^5^ Guizhou Center for Disease Control and Prevention Guiyang China; ^6^ Department of Cardiology, Fuwai Shenzhen Hospital Chinese Academy of Medical Sciences Shenzhen China

**Keywords:** depressive disorder, early prediction, machine learning, type 2 diabetes mellitus, visual function index

## Abstract

Visual impairment has been recognized as a potential risk factor for depressive symptoms (DS) in diabetes patients, yet the role of visual function in predicting DS remains unexplored. This study aims to develop and validate a predictive model for DS risk in type 2 diabetes mellitus (T2DM) patients in community health settings, incorporating a visual function index (VF14). We conducted a cross‐sectional study involving 542 T2DM patients from four community health centers in Guiyang. Univariate and multivariate logistic regressions identified significant predictors, while 10 machine learning algorithms were employed to construct the predictive model. Model performance was assessed using such metrics as receiver operating characteristic curves, accuracy, sensitivity, specificity, F1 score, Brier score, C‐index, calibration curves, and decision curve analysis. A restricted cubic spline (RCS) analysis evaluated the score‐dependent risk profiles between the VF14 and DS. Key predictors included body mass index (BMI), self‐reported glycemic status, age‐related macular degeneration, glycated hemoglobin (HbA1c), and VF14. Among the models, the gradient boosting machine exhibited the robust predictive performance, with an area under the curve of 0.73 and sensitivity of 0.72. The Shapley additive explanations analysis identified VF14, BMI, and HbA1c as the top risk factors. RCS analysis revealed a score‐dependent risk profile between VF14 and DS risk. This study introduces a clinically interpretable tool for early DS risk stratification in T2DM patients, offering potential for improved risk assessment and timely intervention in community health settings.

## Graphical abstract

Visual impairment has been associated with an increased risk of depression in patients with diabetes, yet no studies have explored the role of visual function as a predictor of depression. To develop and validate a predictive model for depression risk in patients with Type 2 Diabetes Mellitus (T2DM) in community health settings, incorporating a visual function index. This cross‐sectional study collected data from 542 T2DM patients managed by four community health centers in Guiyang. Univariate logistic regression identified potential predictors, followed by multivariate logistic regression for feature selection. Ten machine learning algorithms were used to build the predictive model. Model performance was evaluated using receiver operating characteristic (ROC) curves, accuracy, sensitivity, specificity, F1 score, Brier score, C index, calibration curves, and decision curve analysis (DCA). A restricted cubic spline (RCS) assessed the dose–response relationship between the visual function index (VF14) and depression. Five significant predictors were identified through logistic regression: body mass index (BMI), self‐reported glycemic status, macular degeneration, HbA1c, and VF14. Among 10 machine learning models, the Gradient Boosting Machine demonstrated the best predictive performance, with a validation set ROC of 0.73, sensitivity of 0.72, specificity of 0.68, accuracy of 0.69, F1 score of 0.58, Brier score of 0.18, and a C index of 0.73. The model exhibited strong calibration and the highest net benefit in the validation set. SHAP analysis identified VF14, BMI, and HbA1c as the top three risk factors. RCS analysis reveals that as VF14 increases from 3 to 14.5, the risk of depression rises correspondingly. By integrating routine community health check‐up metrics with a visual function index, this study developed a clinically interpretable predictive model for early depression risk in T2DM patients. The model will aid in early depression risk assessment and timely interventions in community health management.

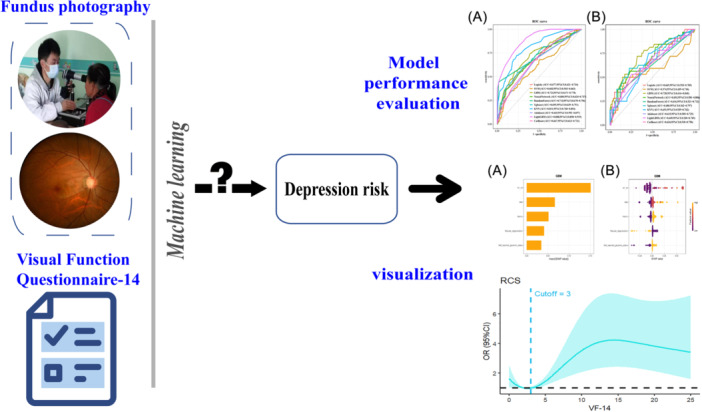

## 1 INTRODUCTION

Diabetes is a prevalent disease characterized by hyperglycemia, relative insulin deficiency, and insulin resistance, posing a significant threat to global public health. According to the latest data from the International Diabetes Federation, the global number of individuals with diabetes has reached 537 million, and this number is projected to increase to 700 million by 2045, with 90% of cases being type 2 diabetes mellitus (T2DM).^1^ Among these patients, approximately 20%–30% experience depressive symptoms (DS), with a prevalence rate 2–3 times higher than that in the general population.^2^ This comorbidity creates a vicious cycle, in which depression exacerbates treatment nonadherence, disrupts the hypothalamic‐pituitary‐adrenal (HPA) axis, and contributes to increased glycemic fluctuations. These factors elevate the risk of microvascular complications, ultimately resulting in higher hospitalization rates and increased all‐cause mortality.^3^ However, early identification of high‐risk groups for depression in T2DM patients remains a significant challenge, particularly in primary care settings.

Community healthcare centers face dual challenges in the management of depression among diabetes patients. First, most T2DM patients rely on primary care facilities for long‐term management,^4^ yet these institutions often lack specialized psychological resources, resulting in insufficient depression screening rates. Second, existing screening tools, such as the Hamilton depression rating scale, focus primarily on the assessment of symptoms but fail to predict the risk of depression. Notably, many primary care centers offer free ophthalmologic screenings for diabetes patients, including fundus photography and visual function assessments. However, much of this valuable data remains underutilized. Research indicates that a 5‐point decrease in the visual function index (VF14) score, reflecting worsening visual impairment and major ocular diseases, is associated with a 40% reduction in patients' social activities.^5^ Furthermore, diabetic retinopathy (DR) patients, due to prolonged visual impairment, experience “disability stress,” which can trigger depression through social isolation and disease‐related stigma.^6^, ^7^ This suggests that visual function may serve as a “bridge biomarker” linking metabolic dysfunction to psychological disorders.

This study focused on the current status of community‐based diabetes management and introduced the VF14 into a predictive model for the first time. Utilizing machine learning algorithms, this model integrates fundus imaging parameters, metabolic indicators, and visual function indices to develop a prediction model for DS risk in T2DM patients, specifically designed for primary healthcare settings.

## 2 METHODS

### 2.1 Study subjects

This cross‐sectional study was conducted between February 2022 and September 2023 across four communities in Guiyang, Guizhou Province, recruiting 655 individuals diagnosed with T2DM. Of these, 615 completed the examination, and data from 542 subjects were included in the final analysis. The inclusion criteria were as follows: (1) age ≥ 18 years; (2) meeting the World Health Organization's (WHO) diagnostic criteria for T2DM (1999); (3) ability to participate in questionnaire surveys and relevant examinations; and (4) clear consciousness and cognitive function. Exclusion criteria included: (1) a history of psychiatric disorders; (2) severe comorbidities such as cancer; (3) intellectual disabilities; (4) history of eye surgery; and (5) conditions obstructing retinal examination due to refractive medium opacities. The study was approved by the Ethics Committee of the Guizhou Provincial Center for Disease Control and Prevention (S2023‑12), and all participants provided written informed consent.

### 2.2 Sample size calculation

Sample size estimation was conducted using R software (version 4.3.3). Based on the reported depression incidence rate of 32.24%^8^ from prior studies, and utilizing the formula for sample size calculation in cross‐sectional studies, the epi.sssimpleestb function from the epir package in R was employed. The parameters were set as follows: side.test = 2, power = 0.80, and confidence level = 0.95. The required sample size was calculated to be 357 participants. Considering a 20% non‐response rate, the final calculated sample size was 447 participants, which was met by the patient recruitment in this study.

### 2.3 Study procedures

#### 2.3.1 Questionnaire survey

A self‐designed questionnaire was administered to gather demographic information, including age, sex, education level, hypertension status, medication usage for diabetes, and disease duration. Trained interviewers conducted face‐to‐face interviews.

#### 2.3.2 Visual function assessment

Visual function was assessed using the Chinese version of the VF14 scale (12‐Item Version), which comprises 12 items covering four domains: near vision (e.g., reading small text, fine manual tasks), visual adaptation (e.g., navigating stairs, reading signs), subjective visual ability (e.g., reading large text, recognizing faces), and stereopsis (e.g., recreational activities, sports). Responses were scored on a 5‐point scale ranging from 0 (“no difficulty”) to 4 (“unable to perform”), with higher scores indicating worse visual function. The total score of the VF14 ranges from 0 to 48 (12 items × 4 points), with the average score (calculated by dividing the total score by the number of items) ranging from 0 to 4. Higher scores indicate poorer visual function. The VF14 demonstrated excellent internal consistency with a Cronbach's alpha of 0.863, indicating robust reliability and validity.^9^ Reliability and validity tests conducted on the VF14 used in this study showed excellent results, with a Cronbach's alpha of 0.927 and a Kaiser‐Meyer‐Olkin value of 0.905, confirming robust psychometric properties.

#### 2.3.3 DS screening

DS were assessed using the Patient Health Questionnaire‐9 (PHQ‐9), consisting of 9 items with a 4‐point response scale. Each item is scored as follows: 0 = “not at all”, 1 = “several days,” 2 = “more than 1 week,” and 3 = “nearly every day.” A total score ≥5 was considered indicative of DS. The PHQ‐9 demonstrated good reliability (Cronbach's *α* = 0.89).^10^

#### 2.3.4 Physical measurements

Height and weight were measured using a SH‐20A height and weight measuring device. Body mass index (BMI) was calculated as weight (kg) divided by height squared (m²). Visual acuity was assessed using a standard logarithmic visual acuity chart, based on national standards (GB_T11533‐2011).^11^

#### 2.3.5 Retinal imaging

Participants underwent retinal imaging using the MOCULAR (ML‐800s) non‐mydriatic fundus camera in a darkened room. Both left and right eye images centered on the macula were obtained. Cases with poor image quality or opacity that obstructed retinal imaging were excluded. Images were reviewed by trained ophthalmologists following the latest clinical guidelines for DR diagnosis.

#### 2.3.6 Laboratory tests

Blood pressure was measured using a Yuwell electronic sphygmomanometer, with hypertension defined as either a self‐reported diagnosis or ongoing antihypertensive treatment, or a systolic blood pressure ≥140 mmHg (18.6 kPa) and/or diastolic blood pressure ≥90 mmHg (12.0 kPa). Glycated hemoglobin (HbA1c) was measured using a Sinocare PCH‐100 portable analyzer.

### 2.4 Statistical analysis

Missing data were handled using multiple imputation via the Multivariate Imputation by Chained Equations (MICE) package. To ensure the stability and accuracy of the imputation results, we set the number of imputations to 5. Each imputation generated a complete dataset, thereby reducing the impact of imputation error on the final analytical results. During the imputation process, variables that could potentially affect the study outcomes were taken into account, including age, sex, BMI, and duration of diabetes. A multivariate regression model was employed as the basic framework for imputation. Specifically, during the data partitioning stage, the entire dataset was imputed first, after which it was divided into training and testing sets.

Continuous variables were expressed as mean ± standard deviation, and categorical variables were presented as frequency and percentage. Group comparisons were conducted using *t*‐tests or Chi‐square tests. All statistical analyses were performed using R software (version 4.3.3), with *p* < 0.05 considered statistically significant.

The dataset was randomly divided into training (*n* = 434) and validation (*n* = 108) sets in an 80:20 ratio using the R random seed generator (seed = 123). Balance tests were performed between the two groups (Table 1). Significant variables from univariate analysis without multicollinearity were included in multivariate logistic regression. Feature selection identified five significant variables. Ten machine learning models, including logistic regression, support vector machine (SVM), gradient boosting machine (GBM), neural networks, random forest, extreme gradient boosting (XGBoost), K‐nearest neighbor (KNN), adaptive boosting (AdaBoost), LightGBM, and categorical boosting (CatBoost), were constructed. A fivefold, 10‐iteration cross‐validation process and grid search were conducted to ensure model stability.

**Table 1:** Comparison of baseline characteristics between the training set and testing set of study subjects.

Variables		Train set (*n* = 434)		*p‐*value	Test set (108)		*p‐*value
		No DS (*n* = 300)	DS (*n* = 134)		No DS (*n* = 79)	DS (*n* = 29)	
Age (years)	<65	115	52	0.93	27	10	0.978
	≧65	185	82		52	19	
Sex	Female	156	74	0.54	50	18	0.91
	Male	144	60		29	11	
BMI	Normal	129	46	0.09	37	10	0.28
	Overweight	142	67		34	13	
	Obesity	29	21		8	6	
Education (years)	<9	177	82	0.67	46	20	0.31
	≧9	123	52		33	9	
Hypertension	Yes	168	70	0.47	40	19	0.17
	No	132	64		39	10	
Vision	<4.9	209	95	0.80	54	17	0.35
	≧4.9	91	39		25	12	
Smoking	Yes	56	29	0.47	16	2	0.10
	No	244	119		63	27	
Drinking	Yes	35	15	0.89	8	2	0.61
	No	265	105		71	41	
Medication adherence	Regular	214	90	0.38	55	18	0.46
	Irregular	86	44		24	11	
Self‐reported glycemic status	Abnormality	62	13	0.01	17	3	0.19
	Normal	238	121		62	26	
AMD	Yes	63	15	0.01	19	5	0.45
	No	237	119		60	24	
Optic disc disorders	Yes	21	7	0.49	4	2	0.71
	No	279	127		75	27	
DR	Yes	97	37	0.33	21	7	0.80
	No	203	97		58	22	
HbA1c	<6.5	137	45	0.02	42	12	0.28
	≧6.5	163	89		37	17	
DM duration (years)	<9	166	69	0.46	45	14	0.42
	≧9	134	65		34	15	
Personal annual income (10,000 yuan)	‐	4.98 ± 3.98	4.99 ± 3.10	0.79	5.13 ± 3.71	5.65 ± 4.47	0.54
VF14	‐	4.31 ± 5.93	6.54 ± 7.23	<0.001	3.73 ± 5.21	6.59 ± 7.29	0.03

Abbreviation: AMD, age‐related macular degeneration; BMI, body mass index; DS, depressive symptoms; DM, diabetes mellitus; DR, diabetic retinopathy; HbA1c, glycated hemoglobin; VF14, visual function index.

Model performance was evaluated using receiver operating characteristic (ROC) curves analysis, with the area under the curve (AUC), accuracy, specificity, sensitivity, F1 score, Brier score, and C index as key metrics. The optimal model was selected based on the highest AUC and accuracy in the validation set. Calibration curves were used to assess the consistency of depression prediction with actual outcomes. Decision curve analysis (DCA) was performed to evaluate clinical net benefit.

**Figure 1:**
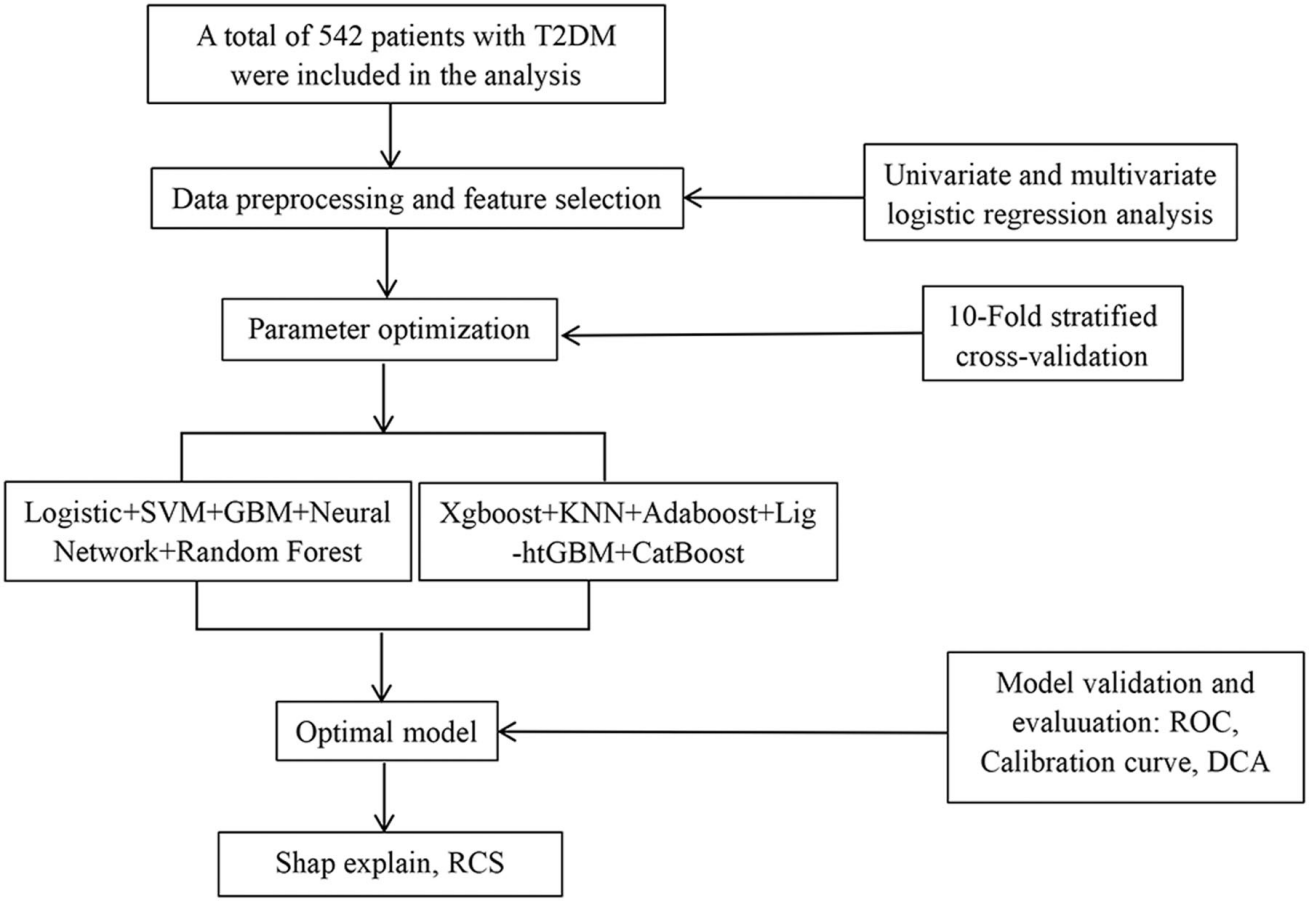
Flowchart of this study. AdaBoost, adaptive boosting; CatBoost, categorical boosting; DCA, decision curve analysis; GBM, gradient boosting machine; KNN, K‐nearest neighbor; SVM, support vector machine; ROC, receiver operating characteristic; RCS, restricted cubic spline; T2DM, type 2 diabetes mellitus; XGBoost, extreme gradient boosting.

The Shapley additive explanations (SHAP) method was employed to interpret the optimal model, providing estimates of each feature's impact on predictions. SHAP significance analysis, summary plots, and dependence plots were used to evaluate feature importance and their effects on outcome predictions. Finally, SHAP force plots and a waterfall chart illustrated the contribution of individual features in specific patients. To explore the potential nonlinear relationship between VF14 and depression risk in T2DM, a restricted cubic spline (RCS) was applied (Figure 1).

## 3 RESULTS

### 3.1 Baseline characteristics

A total of 542 participants were included in the study (training set: *n* = 434; testing set: *n* = 108), with an average age of 66.19 ± 8.91 years. Among the participants, 244 (45.02%) were male and 298 (54.98%) were female. As shown in Table 1, in the training set, significant differences were observed between the DS and non‐DS groups in self‐reported glycemic status (*χ*² = 7.791, *p* = 0.01), age‐related macular degeneration (AMD) (*χ*² = 6.042, *p* = 0.01), and HbA1c ≥ 6.5% (*χ*² = 5.555, *p* = 0.02). In addition, the DS group exhibited significant poorer visual function as measured by the VF‐14 (*t* = −3.386, *p* < 0.001). Notably, in the testing set, only the VF14 score showed a statistically significant difference between the groups (*t* = −1.933, *p* = 0.03). Demographic characteristics (age, gender, BMI), lifestyle factors (smoking, drinking), and metabolic indicators did not show any significant differences between the depressive and non‐depressive groups (*p* > 0.05).

### 3.2 Multivariable logistic regression analysis of DS predictive factors

Table 2 presented the results of both univariate and multivariate logistic regression analyses for the training set. In the multivariate model, obesity (OR = 2.20, 95% CI 1.15–4.21), self‐reported glycemic status (OR = 2.07, 95% CI 1.08–3.99), and HbA1c ≥ 6.5% (OR = 1.62, 95% CI 1.04–2.53) were independently associated with an increased risk of DS. In contrast, AMD was inversely associated with DS (OR = 0.46, 95% CI 0.25–0.86). To further investigate whether the association between AMD and DS could yield unexpected results, we conducted stratified and sensitivity analyses. The results confirmed that the association between AMD and DS remained robust, as shown in Supplementary Tables 1 and 2 (Appendix 1). For each 1‐point increase in the VF14 score, DS increased by 5% (OR = 1.05, 95% CI 1.01‐1.08). Additionally, the maximum variance inflation factor for the covariates in the model was 1.403, and the minimum tolerance was 0.713, indicating no multicollinearity.

**Table 2:** The result of univariate and multivariate logistic regression analysis for training set.

Variables		Univariable		Multivariable	
		OR (95%CI)	*p‐*value	OR (95%CI)	*p‐*value
Age (years)	<65	‐	‐	‐	‐
	≧65	0.98 (0.65–1.49)	0.93	‐	‐
Sex	Female	‐	‐	‐	‐
	Male	1.14 (0.76–1.71)	0.53		
BMI	Normal	‐	‐	‐	‐
	Overweight	1.27 (0.81–1.99)	0.30	1.35 (0.85–2.15)	0.21
	Obesity	2.28 (1.22–4.24)	0.01	2.20 (1.15–4.21)	0.02
Education (years)	<9	‐	‐	‐	‐
	≧9	0.91 (0.60–1.38)	0.67	‐	‐
Hypertension	Yes	‐	‐	‐	‐
	No	0.86 (0.57–1.29)	0.47	‐	‐
Vision	<4.9	‐	‐	‐	‐
	≧4.9	1.06 (0.68–1.66)	0.80	‐	‐
Smoke	Yes	‐	‐	‐	‐
	No	1.20 (0.73–1.99)	0.47	‐	‐
Drink	Yes	‐	‐	‐	‐
	No	0.95 (0.50–1.81)	0.89	‐	‐
Medication adherence	Regular	‐	‐	‐	‐
	Irregular	1.22 (0.78–1.89)	0.38	‐	‐
Self‐reported glycemic status	Abnormality	‐	‐	‐	‐
	Normal	2.42 (1.28–4.58)	0.01	2.07 (1.08–3.99)	0.03
AMD	Yes	‐	‐	‐	‐
	No	0.47 (0.26–0.87)	0.01	0.46 (0.25–0.86)	0.02
Optic disc disorders	Yes	‐	‐	‐	‐
	No	0.73 (0.30–1.77)	0.49	‐	‐
DR	Yes	‐	‐	‐	‐
	No	0.80 (0.51–1.25)	0.33	‐	‐
HbA1c	<6.5	‐	‐	‐	‐
	≧6.5	1.66 (1.09–2.54)	0.02	1.62 (1.04–2.53)	0.03
DM duration (years)	<9	‐	‐	‐	‐
	≧9	1.17 (0.78–1.76)	0.46	‐	‐
Personal annual income (10,000 yuan)	‐	1.00 (0.95–1.06)	0.97	‐	‐
VF14	‐	1.05 (1.02–1.09)	<0.001	1.05 (1.01–1.08)	0.01

Abbreviations: AMD, age‐related macular degeneration; BMI, body mass index; DM, diabetes mellitus; DR, diabetic retinopathy; HbA1c, glycated hemoglobin; VF14, visual function index.

### 3.3 Model construction and comparison

**Figure 2:**
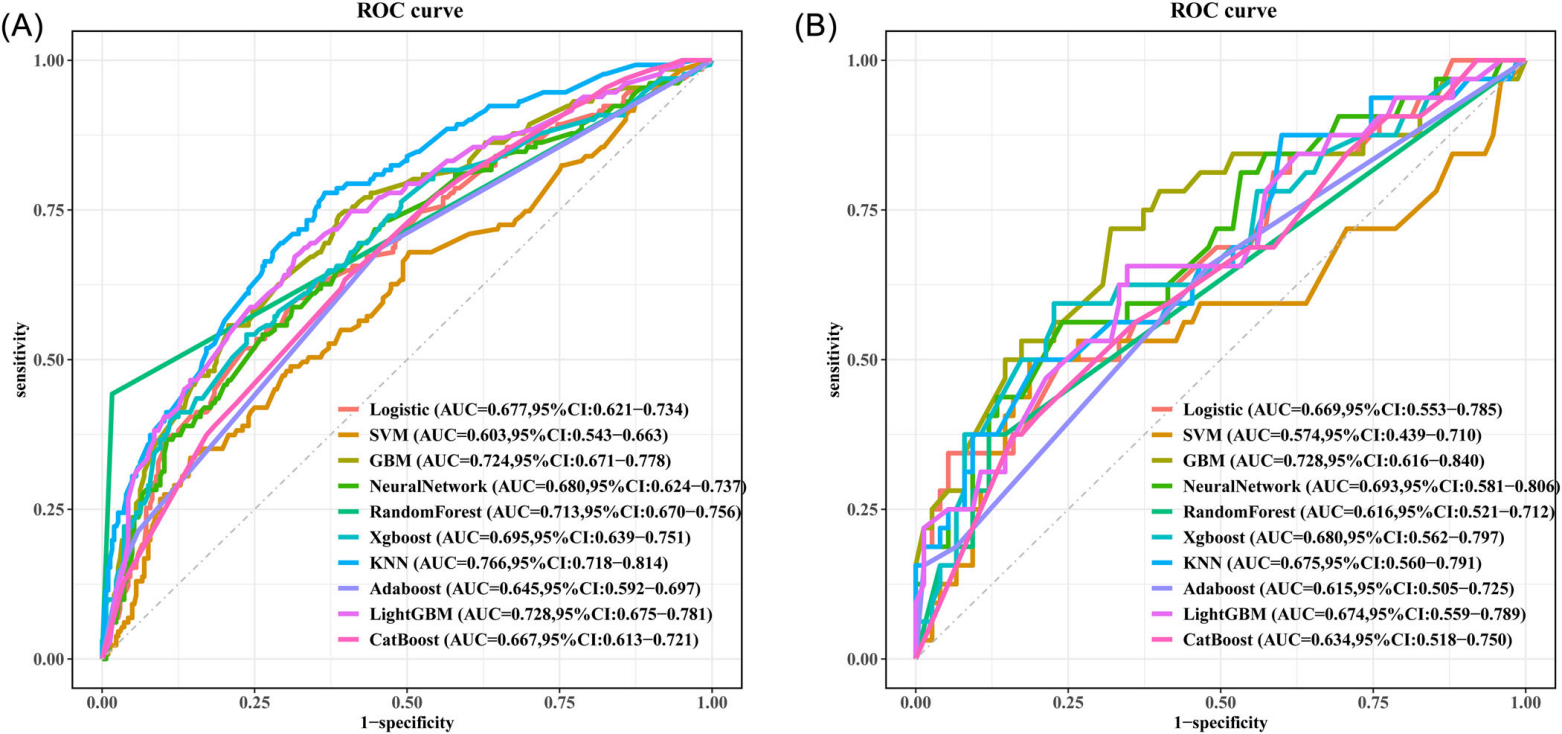
The ROC curve analysis comparison of 10 machine learning algorithms on the training set (A) and the testing set (B). AdaBoost, adaptive boosting; CatBoost, categorical boosting; GBM, gradient boosting machine; KNN, K‐nearest neighbor; SVM, support vector machine; ROC, receiver operating characteristic; XGBoost, extreme gradient boosting.

The performance comparison of various machine learning models is summarized in Table 3 and Figure 2. Table 3 provides detailed metrics, including AUC, accuracy, sensitivity, specificity, F1 score, Brier score, and C index for 10 models. The AUC values of different models in the training set ranged from 0.60 to 0.77. The KNN model exhibited the highest performance with an AUC of 0.77, an accuracy of 0.68, sensitivity of 0.78, specificity of 0.64, F1 score of 0.59, Brier score of 0.17, and a C index of 0.77 (Table 3, Figure 2A). Besides, the GBM model also exhibited a good performance with an AUC of 0.72 (Table 3). Notably, in the test set, the GBM model demonstrated the best performance with an AUC of 0.73 (Figure 2B) and an accuracy of 0.69, surpassing other models in terms of AUC, indicating that GBM provided the best predictive performance. Additionally, we have included the confusion matrix for the GBM model in Appendix 2.

**Table 3:** Comparison of prediction performance of 10 machine learning models on training and validation sets.

Data set	Classification model	AUC (95%CI)	Accuracy	Sensitivity	Specificity	F1‐Score	Brier score	C index
Training set	Logistic	0.68 (0.62–0.73)	0.69	0.52	0.77	0.50	0.19	0.68
	SVM	0.60 (0.54–0.66)	0.70	0.34	0.86	0.40	0.21	0.60
	GBM	0.72 (0.67–0.78)	0.65	0.74	0.61	0.56	0.18	0.72
	Neural Network	0.68 (0.62–0.74)	0.68	0.54	0.74	0.50	0.19	0.68
	Random Forest	0.71 (0.67–0.76)	0.82	0.44	0.98	0.60	0.18	0.71
	Xgboost	0.70 (0.64–0.75)	0.70	0.54	0.76	0.52	0.25	0.70
	KNN	0.77 (0.72–0.81)	0.68	0.78	0.64	0.59	0.17	0.77
	Adaboost	0.65 (0.59–0.70)	0.59	0.69	0.54	0.50	0.22	0.65
	LightGBM	0.73 (0.68–0.78)	0.68	0.67	0.68	0.56	0.19	0.73
	CatBoost	0.67 (0.61–0.72)	0.61	0.63	0.60	0.50	0.28	0.67
Testing set	Logistic	0.67 (0.55–0.79)	0.77	0.34	0.95	0.47	0.19	0.67
	SVM	0.57 (0.44–0.71)	0.72	0.5	0.81	0.52	0.21	0.57
	GBM	0.73 (0.62–0.84)	0.69	0.72	0.68	0.58	0.18	0.73
	Neural Network	0.69 (0.58–0.81)	0.70	0.56	0.76	0.53	0.19	0.69
	Random Forest	0.62 (0.52–0.71)	0.73	0.38	0.88	0.45	0.26	0.62
	Xgboost	0.68 (0.56–0.80)	0.72	0.59	0.77	0.56	0.25	0.68
	KNN	0.68 (0.56–0.79)	0.71	0.5	0.8	0.51	0.19	0.68
	Adaboost	0.62 (0.51–0.73)	0.56	0.66	0.52	0.47	0.23	0.62
	LightGBM	0.67 (0.56–0.79)	0.65	0.66	0.65	0.53	0.19	0.67
	CatBoost	0.63 (0.52–0.75)	0.70	0.38	0.84	0.43	0.28	0.63

Abbreviations: AdaBoost, adaptive boosting; CatBoost, categorical boosting; GBM, gradient boosting machine; KNN, K‐nearest neighbor; SVM, support vector machine; XGBoost, extreme gradient boosting.

**Figure 3:**
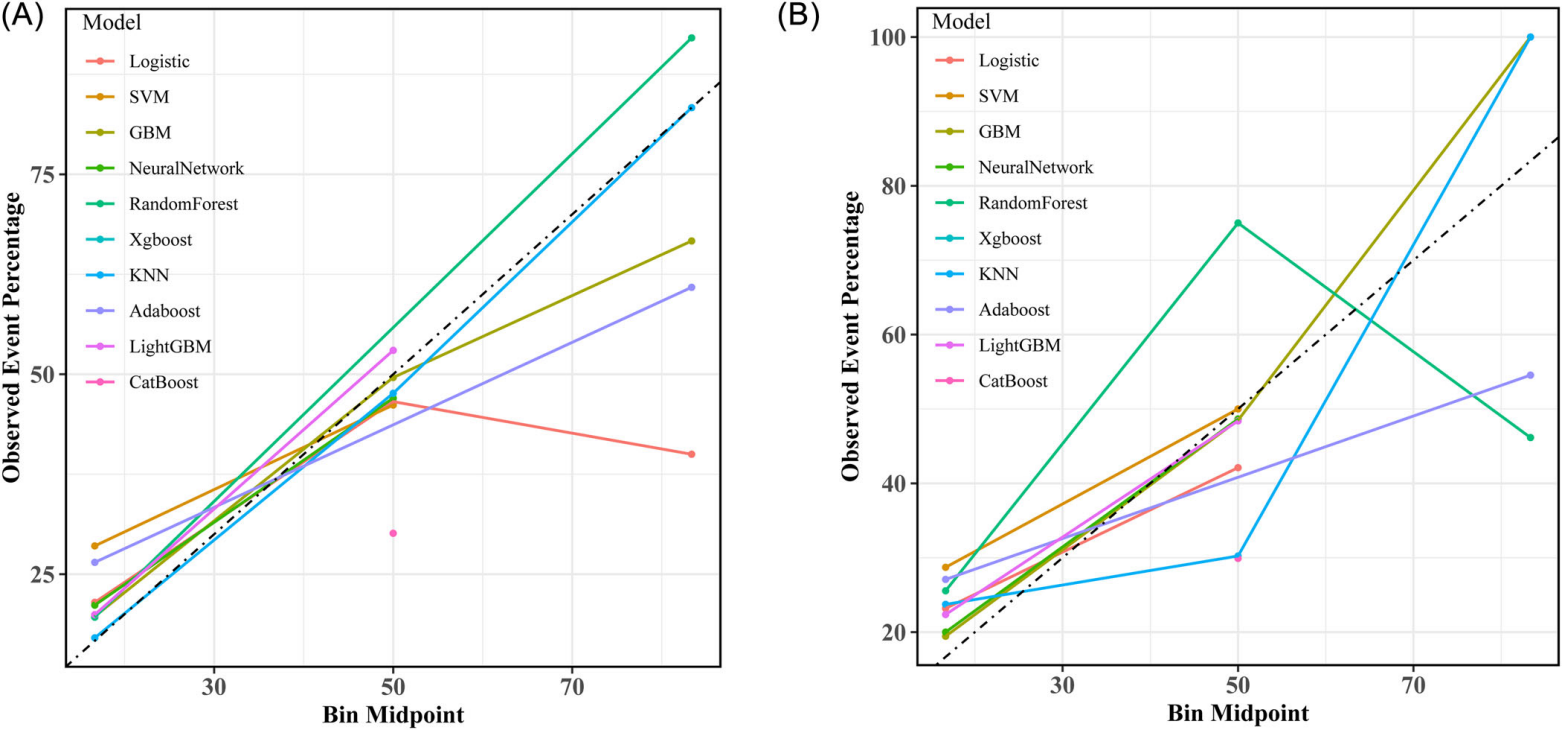
Comparison of the calibration curves of 10 machine learning algorithms on the training set (A) and the testing set (B). AdaBoost, adaptive boosting; CatBoost, categorical boosting; GBM, gradient boosting machine; KNN, K‐nearest neighbor; SVM, support vector machine; XGBoost, extreme gradient boosting.

**Figure 4:**
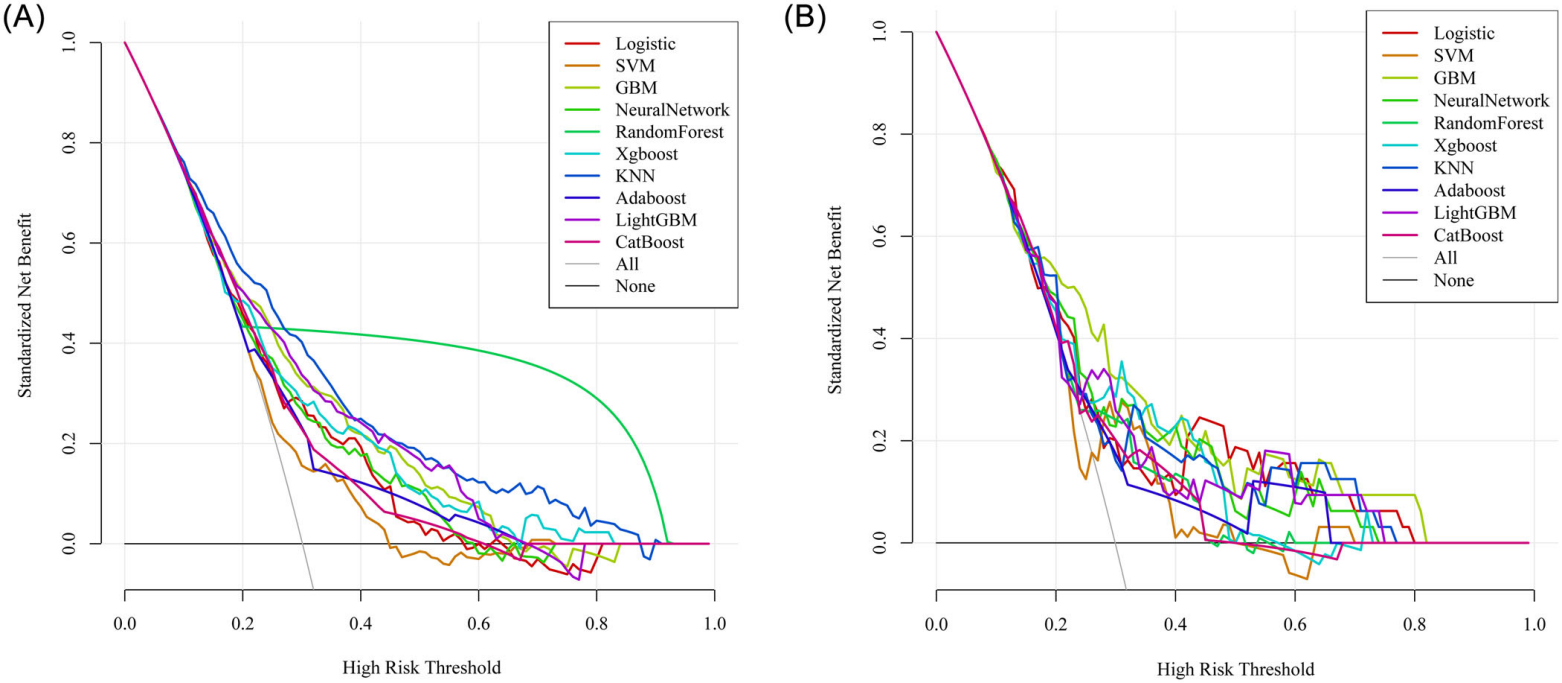
Comparison of the DCA curve analysis of 10 machine learning algorithms on the training set (A) and the testing set (B). AdaBoost, adaptive boosting; CatBoost, categorical boosting; GBM, gradient boosting machine; KNN, K‐nearest neighbor; SVM, support vector machine; XGBoost, extreme gradient boosting.

To assess model calibration, calibration curves were generated and compared. In the training set, KNN exhibited the best fit between observed and predicted probabilities (Figure 3A), while in the test set, GBM showed the best fit between observed and predicted probabilities (Figure 3B). DCA was conducted to evaluate the clinical utility of the models. The results indicated that in the training set, the KNN model provided the highest net benefit in predicting DS (Figure 4A), while in the test set, the GBM model delivered the highest net benefit in predicting DS, at threshold probabilities ranging from 0.18 to 0.81; the GBM model demonstrated higher net benefits in predicting the risk of DS in patients with T2DM. These findings suggest that the GBM model holds significant clinical value across a broad range of threshold probabilities (Figure 4B).

### 3.4 Interpretability analysis based on the SHAP algorithm

**Figure 5:**
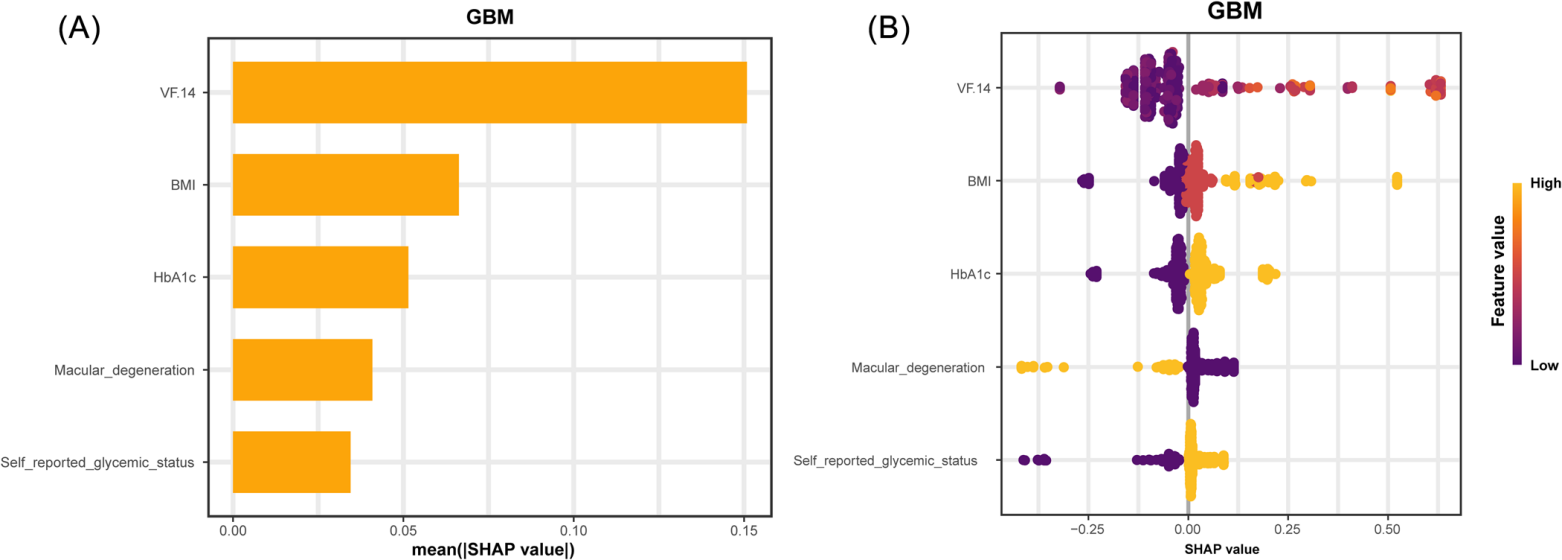
Variable importance ranking (A) and variable contribution (B) from SHAP analysis of the GBM model. BMI, body mass index; GBM, gradient boosting machine; HbA1c, glycated hemoglobin; VF14, visual function index.

The SHAP algorithm was applied to perform an interpretability analysis of the GBM model. The feature importance ranking based on SHAP values revealed that the VF14 score exhibited the strongest predictive value among all predictors, with higher VF14 scores correlating with an increased risk of DS. Other important predictors included BMI and HbA1c (Figure 5A).

The distribution of feature impacts on model outputs illustrated the positive and negative relationships between the predictors and the target outcome (Figure 5B). Points of different colors represented the attribution of results for all patients, with yellow points indicating high‐risk values and purple points indicating low‐risk values. It demonstrated that decreased VF14 value was associated with a lower risk of SD. Increases in BMI and HbA1c positively influenced the predictions, making the outcomes more likely to indicate DS (Figure 5B).

**Figure 6:**
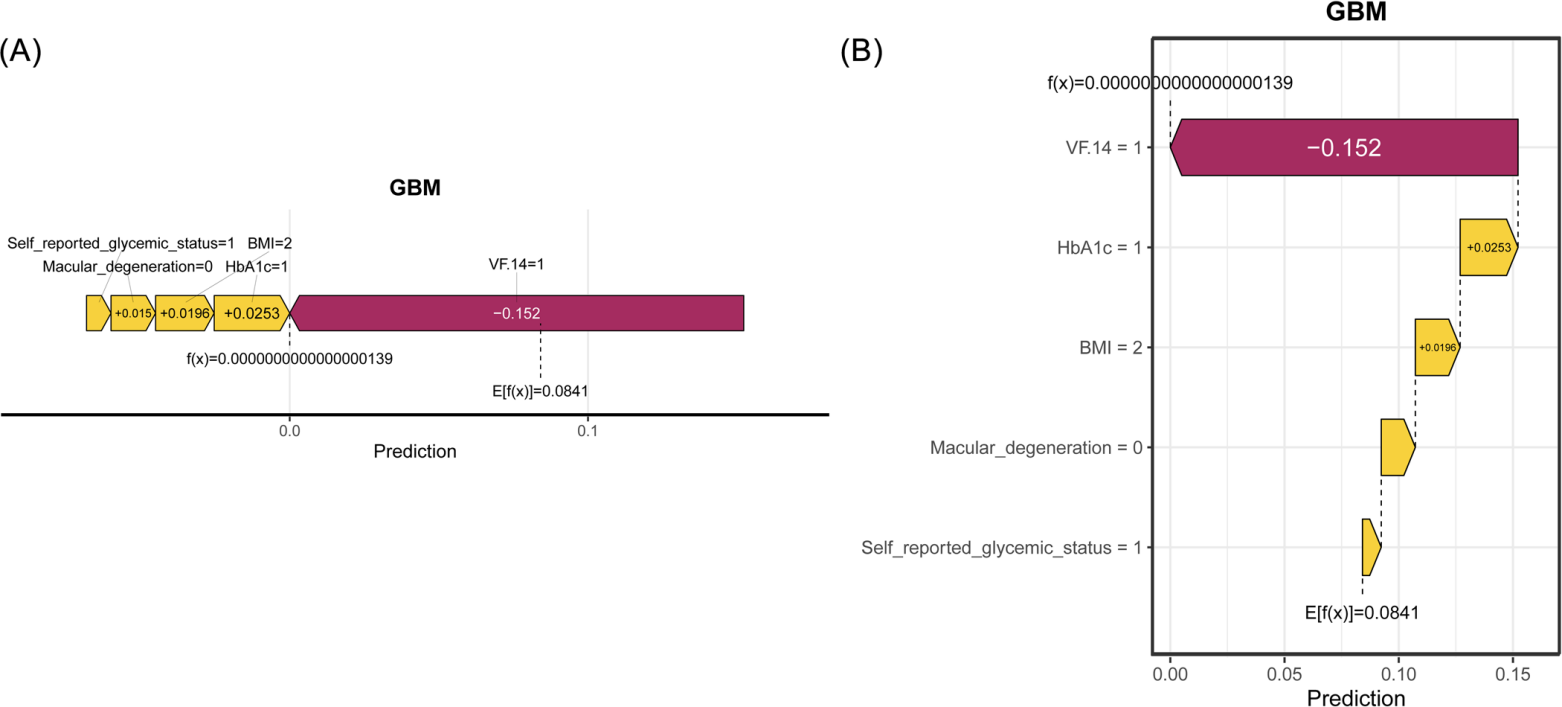
Force plot (A) and waterfall plot (B) used to explain the contribution of features for a specific patient. The yellow and purple bars represent positive and negative contributions to the depression incidence, respectively. BMI, body mass index; GBM, gradient boosting machine; HbA1c, glycated hemoglobin; VF14, visual function index.

Additionally, we visualized the SHAP values for individual patients using force plots (Figure 6A) and waterfall charts (Figure 6B). These visualizations provide clinicians with a rapid understanding of the factors most closely associated with either an increase or a decrease in a patient's DS risk.

### 3.5 Score‐dependent risk profiles between VF14 and depression

**Figure 7:**
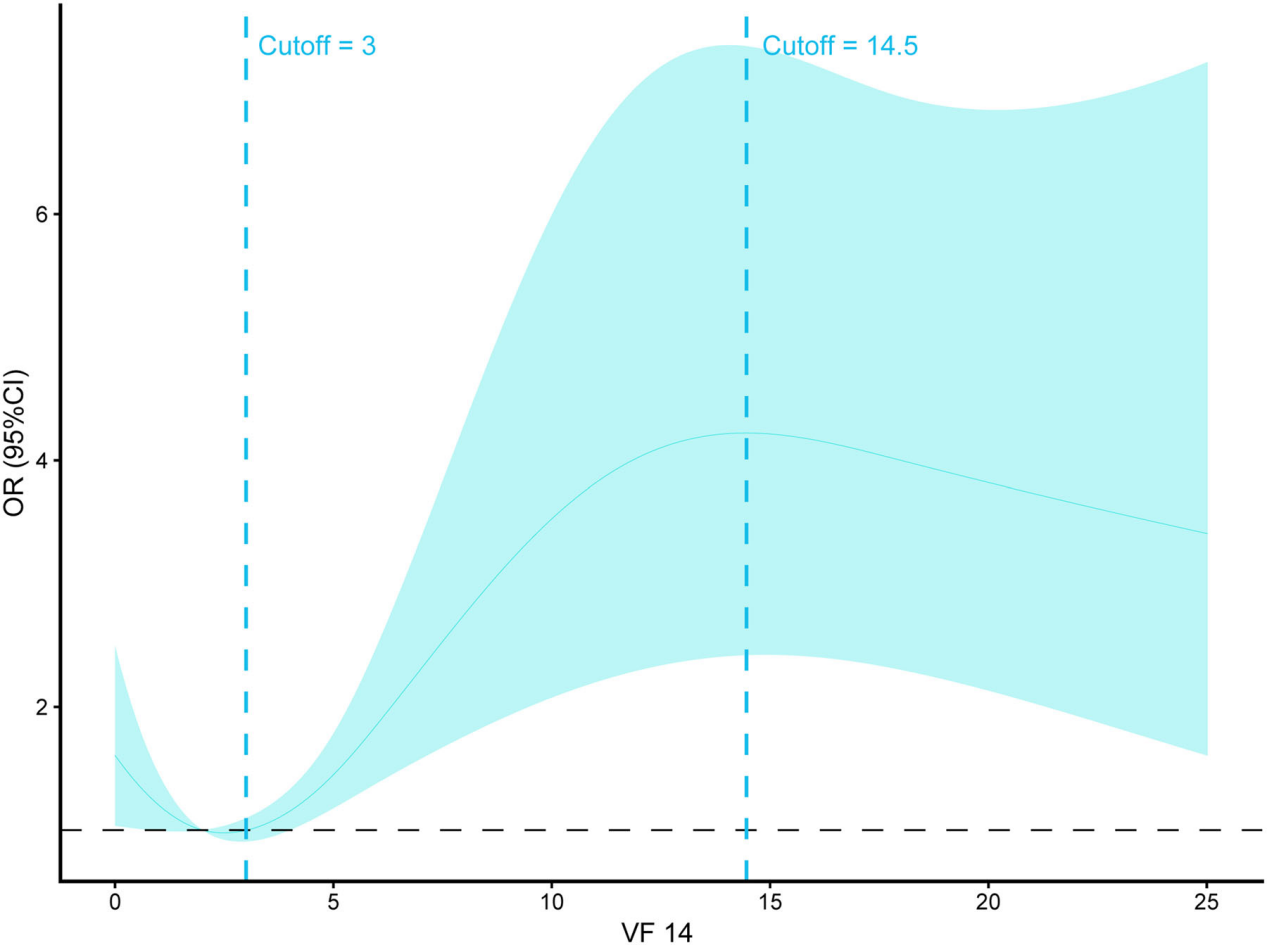
Restricted cubic spline plot of depression and VF14.

The previous results indicated a positive correlation between VF14 scores and DS. To further explore the score‐dependent risk profiles, this study employed RCS analysis. The RCS results revealed an S‐shaped curve between VF14 and DS risk in patients with T2DM. DS risk was lowest when VF14 was 3. The risk of depression remained relatively stable or slightly decreased at lower levels of visual impairment, reaching a local minimum at a cutoff of 3 points. Beyond this threshold, the OR for depression exhibited a sharp, steep increase as VF‐14 scores rose, reaching its peak at a cutoff of 14.5 points. Beyond 14.5, the OR curve showed a slight downward trend, although the risk remained significantly higher than the baseline. The confidence intervals did not include an OR of 1, confirming the statistical significance of this nonlinear relationship (*p* non‐linearity < 0.001) (Figure 7).

## 4 DISCUSSION

To our knowledge, this was the first to incorporate the VF14 scale in constructing a DS risk prediction model for patients with T2DM in community health service settings. Multiple validation methods indicated that the model demonstrated robust predictive performance and substantial clinical value. By comparing the performance of 10 machine learning models, we selected the GBM algorithm as the most optimal for building the DS risk prediction model for T2DM patients in community management. The model was further evaluated and validated through fivefold cross‐validation, calibration, and clinical efficacy assessments. The results confirmed that the GBM‐based prediction model for DS in T2DM patients in community management has moderate discrimination. This model serves as a risk stratification tool aimed at identifying high‐risk groups and providing a basis for subsequent interventions.

Among all the predictors for DS in diabetic populations, the VF14 score emerged as the most significant. When VF14 exceeds 3, DS risk increases in direct proportion to the VF14 score. This finding provides empirical support for the hypothesis that visual dysfunction contributes to depression via the pathway of social function deprivation.^7^ This aligns with previous studies that reported associations between vision impairment and depression in elderly populations.^12^ However, this study uniquely quantifies the predictive efficacy of visual function for depression risk in a community‐managed T2DM cohort. Visual processing ability in depressive patients is often impaired. Previous research has identified significant deficits in contrast sensitivity, visual masking, and facial expression recognition among depressed individuals, which may exacerbate anhedonia through suppression of reward pathways. Neuroimaging studies suggest that abnormal functional connectivity in visual cortices, the amygdala, and reward‐related brain regions may underpin these deficits.^13^, ^14^ Salmela et al. found that depressed patients have significantly reduced sensitivity to image contrast compared to healthy individuals, a difference linked to abnormal cortical information processing, suggesting functional alterations in the visual system of depressive patients.^15^ There is a bidirectional relationship between visual impairment and depression, where visual deficits can trigger depression, and depression, in turn, exacerbates visual dysfunction. Research by Wu et al. indicated that self‐reported vision decline in populations with visual cortex malfunction—particularly the elderly—correlates with a significantly elevated depression risk, likely due to limitations in social activity and daily functioning.^16^ Rovner et al. also proposed that the inability to engage in meaningful activities could increase the risk of depression.^17^ Depressed patients often experience subjective visual disturbances, such as decreased light sensitivity and color distortion,^18^, ^19^ consistent with their clinical manifestation of a “gray world.”^14^ The VF14 scale, by measuring the functional limitations caused by vision impairment in daily activities (e.g., reading, driving, and facial recognition), effectively reflects the degree of visual dysfunction caused by DR and other complications. Its reliance on routine community‐based retinal screenings and vision tests (e.g., reading ability, staircase recognition) eliminates the need for specialized psychological resources or complex equipment. Compared to scales like PHQ‐9 and HAMD, which require additional interviews, VF14 leverages existing healthcare processes, making it particularly suitable for resource‐limited primary healthcare settings. Furthermore, its multidimensional structure, encompassing near vision, visual adaptation, subjective visual experience, and stereopsis, captures the full spectrum of the pathophysiological and social functional impacts of DR.

DR was not associated with the onset of DS; however, it was significantly correlated with levels of HbA1c. Our findings aligned with those of Rees et al., who found that the depression rate among DR patients could reach 15.4%, although no direct link between DR and anxiety or depression was identified.^20^ A separate 4‐year prospective study concluded that depression and anxiety scores did not increase with the progression of diabetes or worsening DR severity.^21^ However, Morjaria et al. found that DR patients had lower anxiety scores than healthy controls due to a residual population of photosensitive retinal ganglion cells remaining unaffected by DR.^22^ Genetic studies have also examined causal relationships between DR and depression or anxiety, suggesting no causal link in European populations.^23^ AMD and the occurrence of DS in the diabetic population represent a potential, hypothesis‐generating association, which is consistent with the findings of Robert et al.^5^ However, we did not account for the influence of some major unmeasured confounders, such as pain, sleep quality, medication use, and social support. This lack of consideration of key unmeasured confounders, coupled with the differences in study populations between our research and that of Robert et al., means that this association does not imply causality but is merely an observational correlation. Future validation through larger sample size cross‐sectional and cohort studies is needed. The synergistic effects of HbA1c and obesity highlight the importance of the “metabolic‐inflammation‐depression” pathway. Hyperglycemia exacerbates inflammation, contributing to the onset of depression.^24^ Excessive fat accumulation activates adipocytes to release inflammatory mediators such as interleukin‐6 (IL‐6) and C‐reactive protein (CRP),^25^ with higher levels of visceral fat inducing metabolic inflammation.^26^, ^27^ Obesity increases pro‐inflammatory cytokines, with CRP and IL‐6 being associated with elevated depression risk.^28^ These findings emphasize the potential relationship between inflammation and depression. In our study, factors such as age, gender, ethnicity, vision, and disease duration did not correlate with depression symptoms, in line with Hayman et al.'s research, which suggested that companionship is a more significant factor than other variables.^12^

This study has several limitations. First, due to its cross‐sectional design, it cannot establish a causal relationship between visual function and depression; prospective cohort studies are needed to explore causality. Second, the study sample consisted of middle‐aged and elderly individuals, and it remains uncertain whether the observed relationship between visual function and DS holds true for other age groups of T2DM patients. Further research is needed to address this question. This was a cross‐sectional study from a single region, and it has not been externally validated. Although the model has certain utility in community screening, the primary role of this tool is for preliminary screening and risk stratification, rather than for final diagnosis. Its performance in clinical applications still requires further validation. Additionally, the study did not fully control for or describe several potential confounders that may influence the relationship between visual function and depression. Factors such as pain, sleep quality, comorbidities, neuropathy, functional status, social support, medication use (e.g., antidepressants or insulin), psychiatric history, and significant life events could all affect both visual function and DS. Future studies should consider these confounding variables during the design phase and adjust for them to enhance the accuracy and reliability of the findings.

## 5 CONCLUSION

We developed a machine learning model using community screening data to assess the depression risk in patients with T2DM. The model demonstrated moderate discrimination and served as a risk stratification tool rather than a diagnostic tool, enabling community doctors to use it to identify T2DM patients with DS. This has the potential to reduce the risk of DS in T2DM patients and significantly improve their prognosis.

## AUTHOR CONTRIBUTIONS

Yuan‐dong Hu, Jun‐hua Wang, and Xin‐lin Luo formulated the initial idea for the real‐world data study design. Rong‐song Sun, Sheng‐xu Huo, Tian‐lin Zhang, and Yi Yang drafted the manuscript and conducted the analysis. Hao‐jiang Ying and Li‐na Xu contributed to the study design by refining the analytical methods and determining appropriate sample selection for both the main and validation analyses, and statistical analysis. All authors have read, revised, and approved the final version of the manuscript.

## CONFLICT OF INTEREST STATEMENT

The authors declare that the research was conducted in the absence of any commercial or financial relationships that could be construed as a potential conflict of interest.

## ETHICS STATEMENT

This study was approved by the Ethics Committee of the Guizhou Provincial Center for Disease Control and Prevention (S2023‐12). All participants provided informed consent prior to participating in the study.

## Supporting information

Appendix 1.

Appendix 2.

## Data Availability

The data used and/or analyzed during the current study are available from the corresponding author upon reasonable request.
