# Supplementary material for: Development of a predictive model for depressive symptoms in type 2 diabetes mellitus patients under community management: Based on visual function index
Source: Ibrain. 2026 Feb 12;12(1):123–36. doi: 10.1002/ibra.70014 (PMC13097430; doi:10.1002/ibra.70014)
Supplement: Supplementary file 1 — Appendix 1. [file IBRA-12-123-s002.docx]

Supplementary Table 1. Stratified analysis of the risk of depression associated with age-related macular degeneration, adjusted for various confounding factors

| Stratification | AMD | Non-depressive | Depressive | OR (95%CI) | χ^2^ | *p* value |
| --- | --- | --- | --- | --- | --- | --- |
| Male | Yes | 37 (21.4%) | 11 (15.5%) | 0.67 (0.32-1.41) | 1.11 | 0.29 |
|  | No | 136 (78.6%) | 60 (84.5%) |  |  |  |
| Female | Yes | 45 (21.8%) | 9 (9.8%) | 0.39 (0.18-0.83) | 6.24 | 0.01 |
|  | No | 161 (78.2%) | 83 (90.2) |  |  |  |
| Total | Yes | 82 (21.6%) | 20 (12.3%) | 0.51 (0.30-0.86) | 6.55 | 0.01 |
|  | No | 297 (78.4%) | 143 (87.7%) |  |  |  |
| <65 years | Yes | 26 (18.3%) | 6 (9.7%) | 0.48 (0.19-1.23) | 2.43 | 0.12 |
|  | No | 116 (81.7%) | 56 (90.3%) |  |  |  |
| ≧65 years | Yes | 56 (23.6%) | 14 (13.9%) | 0.52 (0.28-0.99) | 4.11 | 0.04 |
|  | No | 181 (76.4%) | 87 (86.1%) |  |  |  |
| Total | Yes | 82 (21.6%) | 20 (12.3%) | 0.51 (0.30-0.86) | 6.55 | 0.01 |
|  | No | 297 (78.4%) | 143 (87.3%) |  |  |  |
| Normal | Yes | 36 (21.7%) | 5 (8.9%) | 0.35 (0.13-0.95) | 4.53 | 0.03 |
|  | No | 130 (78.3%) | 51 (91.1%) |  |  |  |
| Overweight | Yes | 40 (22.7%) | 11 (13.8%) | 0.54 (0.26-1.12) | 2.78 | 0.10 |
|  | No | 136 (77.3%) | 69 (86.3%) |  |  |  |
| Obesity | Yes | 6 (16.2%) | 4 (14.8%) | 0.90 (0.23-3.56) | 0.02 | 0.88 |
|  | No | 31 (83.8%) | 23 (85.2%) |  |  |  |
| Total | Yes | 82 (21.6%) | 20 (12.3%) | 0.51 (0.30-0.86) | 6.55 | 0.01 |
|  | No | 297 (78.4%) | 143 (87.7%) |  |  |  |
| Education level <9 years | Yes | 53 (23.8%) | 16 (15.7%) | 0.60 (0.32-1.11) | 2.73 | 0.10 |
|  | No | 170 (76.2%) | 86 (84.3%) |  |  |  |
| Education level ≧9 years | Yes | 29 (18.6%) | 4 (6.6%) | 0.31 (0.10-0.92) | 4.92 | 0.03 |
|  | No | 127 (81.4%) | 57 (93.4%) |  |  |  |
| Total | Yes | 82 (21.6%) | 20 (12.3%) | 0.51 (0.30-0.86) | 6.55 | 0.01 |
|  | No | 297 (78.4%) | 143 (87.7%) |  |  |  |
| Smoker | Yes | 14 (19.4%) | 5 (16.1%) | 0.80 (0.26-2.44) | 0.16 | 0.69 |
|  | No | 58 (80.6%) | 26 (83.9%) |  |  |  |
| Non-smoker | Yes | 68 (22.1%) | 15 (11.4%) | 0.45 (0.25-0.82) | 7.01 | 0.01 |
|  | No | 239 (77.9%) | 117 (88.6%) |  |  |  |
| Total | Yes | 82 (21.6%) | 20 (12.3%) | 0.51 (0.30-0.86) | 6.55 | 0.01 |
|  | No | 297 (78.4%) | 143 (87.7%) |  |  |  |
| Alcohol consumption | Yes | 13 (30.2%) | 2 (11.8%) | 0.77 (0.58-1.02) | 2.22 | 0.14 |
|  | No | 30 (69.8%) | 15 (88.2%) |  |  |  |
| No alcohol consumption | Yes | 69 (20.5%) | 18 (12.3%) | 0.54 (0.31-0.95) | 4.63 | 0.03 |
|  | No | 267 (79.5%) | 128 (87.7%) |  |  |  |
| Total | Yes | 82 (21.6%) | 20 (12.3%) | 0.51 (0.30-0.86) | 6.55 | 0.01 |
|  | No | 297 (78.4%) | 143 (87.7%) |  |  |  |
| Hypertension | Yes | 43 (20.7%) | 11 (12.4%) | 0.54 (0.27-1.11) | 2.90 | 0.09 |
|  | No | 165 (79.3%) | 78 (87.6%) |  |  |  |
| No hypertension | Yes | 39 (22.8%) | 9 (12.2%) | 0.47 (0.21-1.03) | 3.72 | 0.05 |
|  | No | 132 (77.2%) | 65 (87.8%) |  |  |  |
| Total | Yes | 82 (21.6%) | 20 (12.3%) | 0.51 (0.30-0.86) | 6.55 | 0.01 |
|  | No | 297 (78.4%) | 143 (87.7%) |  |  |  |
| Disease duration  <9 years | Yes | 40 (19.0%) | 8 (9.6%) | 0.46 (0.20-1.02) | 3.79 | 0.05 |
|  | No | 171 (81.0%) | 75 (90.4%) |  |  |  |
| Disease duration  ≧9 years | Yes | 42 (25.0%) | 12 (15.0%) | 0.84 (0.70-0.99) | 3.18 | 0.07 |
|  | No | 126 (75.0%) | 68 (85.0%) |  |  |  |
| Total | Yes | 82 (21.6%) | 20 (12.3%) | 0.51 (0.30-0.86) | 6.55 | 0.01 |
|  | No | 297 (78.4%) | 143 (87.7%) |  |  |  |

Supplementary Table 2. Sensitivity analysis of the risk of depression associated with macular degeneration in patients with type 2 diabetes

|  | Model Ⅰ | |  | Model Ⅱ | |  | Mode Ⅲ | |
| --- | --- | --- | --- | --- | --- | --- | --- | --- |
|  | OR | 95%CI |  | OR | 95%CI |  | OR | 95%CI |
| Macular degeneration | 0.507 | 0.299-0.899 |  | 0.508 | 0.299-0.865 |  | 0.478 | 0.264-0.866 |

Note: Model I: Unadjusted; Model II: Adjusted for age, gender, and BMI; Model III: Adjusted for age, gender, BMI, education level, smoking, alcohol consumption, hypertension, visual acuity, diabetes duration, diabetic retinopathy (DR), HbA1c, and income.
