# Supplementary material for: Development of a predictive model for depressive symptoms in type 2 diabetes mellitus patients under community management: Based on visual function index
Source: Ibrain. 2026 Feb 12;12(1):123–36. doi: 10.1002/ibra.70014 (PMC13097430; doi:10.1002/ibra.70014)
Supplement: Supplementary file 2 — Appendix 2. [file IBRA-12-123-s001.docx]

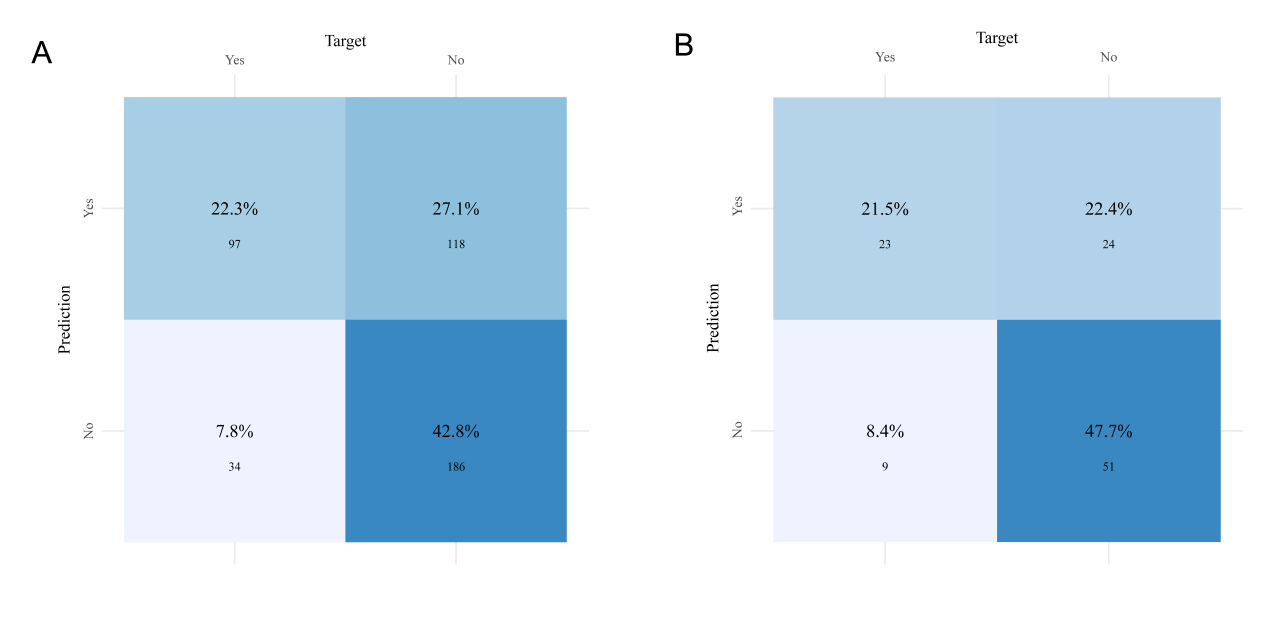


Appendix 2. Confusion matrix for the GBM model. (A). Confounding matrix for the training set; (B). Confounding matrix for the test set.
